# Cardiovascular and renal effects of carperitide and nesiritide in cardiovascular surgery patients: a systematic review and meta-analysis

**DOI:** 10.1186/cc10519

**Published:** 2011-10-27

**Authors:** Chieko Mitaka, Toshifumi Kudo, Go Haraguchi, Makoto Tomita

**Affiliations:** 1 Department of Critical Care Medicine, Tokyo Medical and Dental University Graduate School, 1-5-45, Yushima, Bunkyo-ku, Tokyo, 113-8519, Japan; 2 Intensive Care Unit, Tokyo Medical and Dental University Hospital Faculty of Medicine, 1-5-45, Yushima, Bunkyo-ku, Tokyo, 113-8519, Japan; 3 Clinical Research Center, Tokyo Medical and Dental University Hospital Faculty of Medicine, 1-5-45, Yushima, Bunkyo-ku, Tokyo, 113-8519, Japan

**Keywords:** acute kidney injury, atrial natriuretic peptide, B-type (or Brain) natriuretic peptide, cardiovascular surgery, heart failure, renal function

## Abstract

**Introduction:**

Acute kidney injury (AKI) following cardiovascular surgery is a common disease process and is associated with both morbidity and mortality. The aim of our study was to evaluate the cardiovascular and renal effects of an atrial natriuretic peptide (ANP, carperitide) and a B-type (or brain) natriuretic peptide (BNP, nesiritide) for preventing and treating AKI in cardiovascular surgery patients.

**Methods:**

Electronic databases, including PubMed, EMBASE and references from identified articles were used for a literature search.

**Results:**

Data on the infusion of ANP or BNP in cardiovascular surgery patients was collected from fifteen randomized controlled trials and combined. The infusion of ANP or BNP increased the urine output and creatinine clearance or glomerular filtration rate, and reduced the use of diuretics and the serum creatinine levels. A meta-analysis showed that ANP infusion significantly decreased peak serum creatinine levels, incidence of arrhythmia and renal replacement therapy. The meta-analysis also showed that ANP or BNP infusion significantly decreased the length of ICU stay and hospital stay compared with controls. However, the combined data were insufficient to determine how ANP or BNP infusion during the perioperative period influences long-term outcome in cardiovascular surgery patients.

**Conclusions:**

The infusion of ANP or BNP may preserve postoperative renal function in cardiovascular surgery patients. A large, multicenter, prospective, randomized controlled trial will have to be performed to assess the therapeutic potential of ANP or BNP in preventing and treating AKI in the cardiovascular surgical setting.

## Introduction

Even with the latest advances in surgical and anesthetic techniques and postoperative intensive care, patients undergoing cardiovascular surgery for left ventricular dysfunction still have a fairly high mortality [1,2]. The mortality for severe left ventricular dysfunction in patients undergoing coronary artery bypass grafting is 4.6% to 5.6% and 22.3% to 31% at 30-days and 5 years, respectively, whereas the mortality for normal left ventricular function is 1.1% to 1.9% and 5.5% to 7% at 30 days and 5 years, respectively[1,2]. These cardiovascular surgery patients spend prolonged periods in the ICU and hospital, and their survival is strongly dependent on their pre-operative left ventricular ejection fraction [1]. Cardiorenal syndrome is another serious concern, as patients with left ventricular dysfunction also tend to suffer from acute kidney injury (AKI) [3]. AKI is a common complication in all patients undergoing cardiovascular surgery. A small percentage (0.7% to 3.4%) of patients who develop AKI after cardiovascular surgery require renal replacement therapy, and renal replacement therapy-dependent AKI is an independent risk factor for early mortality following cardiovascular surgery [4-6]. To make matters worse, AKI considerably increases postoperative costs following cardiovascular surgery [7]. The associated mortality risk is additive in non-overlapping patients groups; the hazard ratio for AKI only is 1.41 and for left ventricular dysfunction only is 1.71. In patients in whom both are present, hazard ratio is 3.23 [4]. In any case, interventions to prevent or improve AKI during the perioperative period are clearly needed for cardiovascular surgery patients.

Atrial natriuretic peptide (ANP), a 28-amino-acid peptide hormone synthesized by the cardiac atria, is a potent natriuretic, diuretic, and vasorelaxant substance [8]. ANP increases the pressure within the glomerular capillaries by dilating afferent renal arterioles and constricting efferent renal arterioles [9], resulting in an increase in glomerular filtration. ANP has also been shown to improve renal function in animal models of acute ischemic renal failure [10,11]. B-type (or brain) natriuretic peptide (BNP), a 32-amino-acid peptide initially isolated from porcine brain, was identified as a hormone primarily derived from the cardiac ventricles [12]. The synthesis, secretion and clearance of ANP are different from those of BNP, suggesting discrete pathophysiological roles of ANP and BNP in a dual natriuretic peptide system. ANP is secreted in response to an acute increase in atrial stretch and/or pressure, while BNP is regulated at the gene expression level and responds to increased atrial/ventricular pressure [13,14]. The plasma half-life of ANP is 3 minutes and that of BNP is 20 minutes.

Two synthetic natriuretic peptides are available for use as therapeutic agents in patients with acute heart failure: recombinant human ANP, carperitide (HANP^®^, Daiichisankyo Co., Ltd, Tokyo, Japan) and recombinant human BNP, nesiritide (Natrecor^®^, Scios Inc., Sunnyvale, CA, USA). ANP (carperitide) is commercially available in Japan and has been used in the treatment of acute heart failure. BNP (nesiritide) is available in USA, Switzerland, Argentina, Columbia and Israel [15,16] and has been used in the treatment of fluid-overloaded patients with acute heart failure [17,18].

With these points in mind, we reviewed the literature on the cardiovascular and renal effects of ANP [19] and BNP in cardiovascular surgery patients in order to gain insight into potential postoperative treatment for AKI.

## Materials and methods

We report our study's findings in accordance with the criteria of the PRISMA Group [20]. Electronic databases, including PubMed, EMBASE and references from identified articles between January 1994 and January 2011 were used for a literature search relating to the effects of ANP or BNP on hemodynamics and renal function in cardiovascular surgery patients. The following key words were searched: atrial natriuretic peptide or carperitide, B-type (or brain) natriuretic peptide or nesiritide, acute kidney injury or acute renal failure, left ventricular dysfunction or heart failure, cardiac surgery or cardiovascular surgery. The methodological quality of each study was assessed. Studies were excluded if they were duplicated studies, reviews, retrospective studies, not cardiovascular surgery, not infusion of ANP or BNP, and did not use a control group. We have evaluated the funding source of the individual studies.

### Statistical analysis

Concerning meta-analysis, we calculated the odds ratio (OR) and 95% confidence intervals (CI) between the ANP or BNP group and the control group for need for hemodialysis, incidence of arrhythmias, and mortality. We also calculated weighted difference in means and 95% CI between the ANP or BNP group and the control group for peak serum creatinine levels and length of ICU stay and hospital stay, by the general variance-based method.

## Results

**Figure 1:**
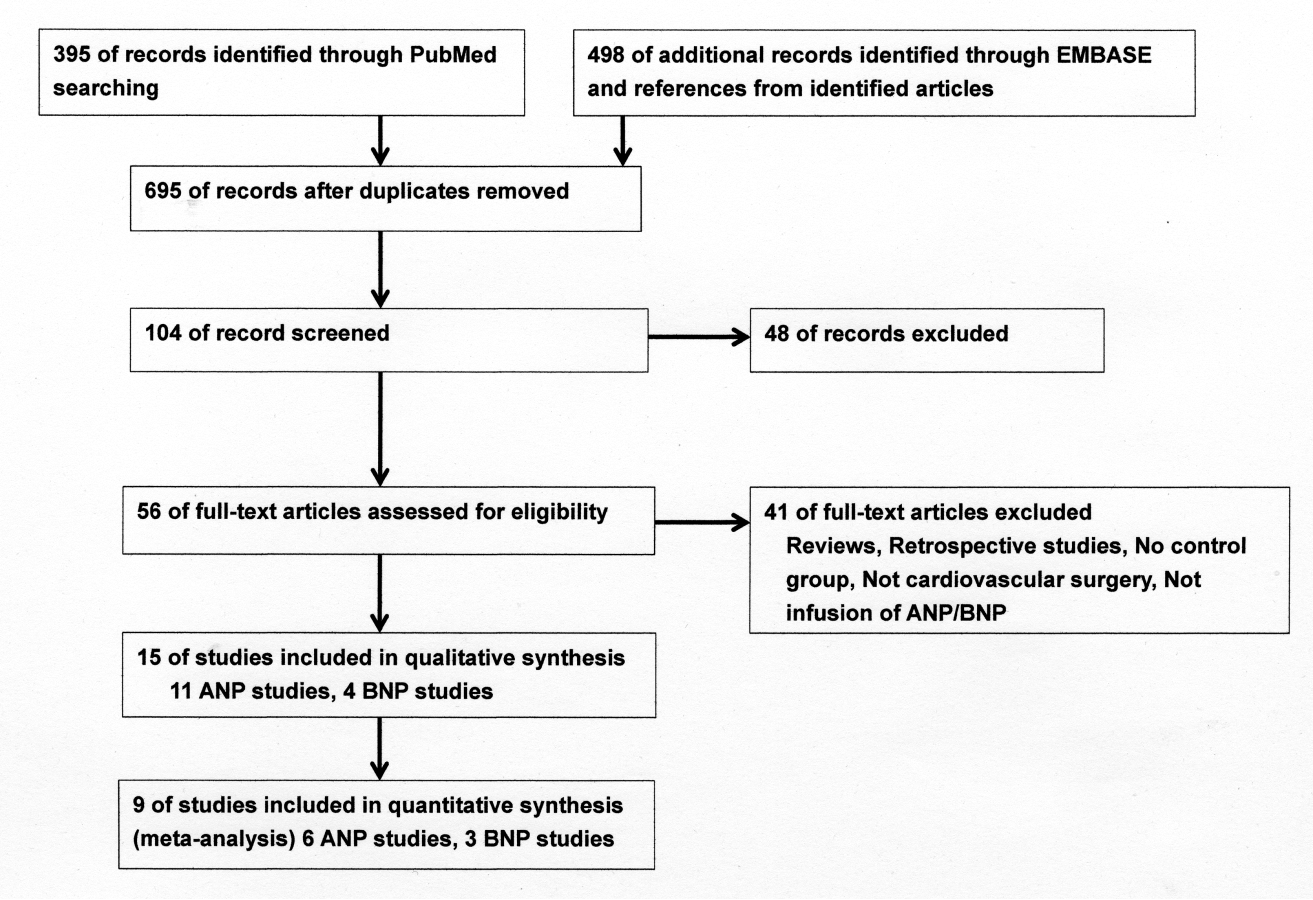
**Flow diagram of study selection**. ANP, atrial natriuretic peptide; BNP, B-type (or brain) natriuretic peptide.

**Figure 2:**
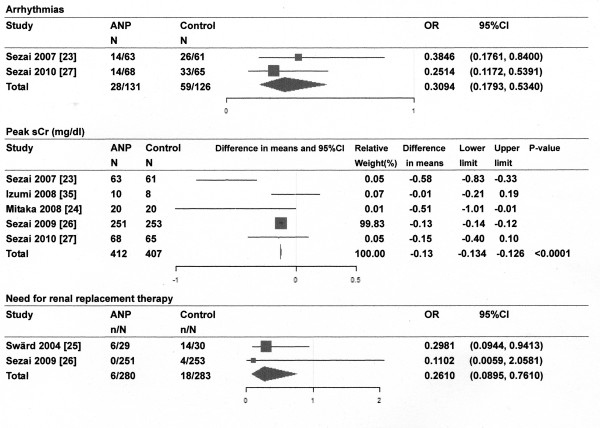
**Effect of atrial natriuretic peptide (ANP) infusion on arrhythmias, peak serum creatinine (sCr) levels and need for renal replacement therapy**. Size of data markers is proportional to the weight of each study in the forest plot. N, number of patients; Horizontal bars = 95% confidence interval (CI).

**Figure 3:**
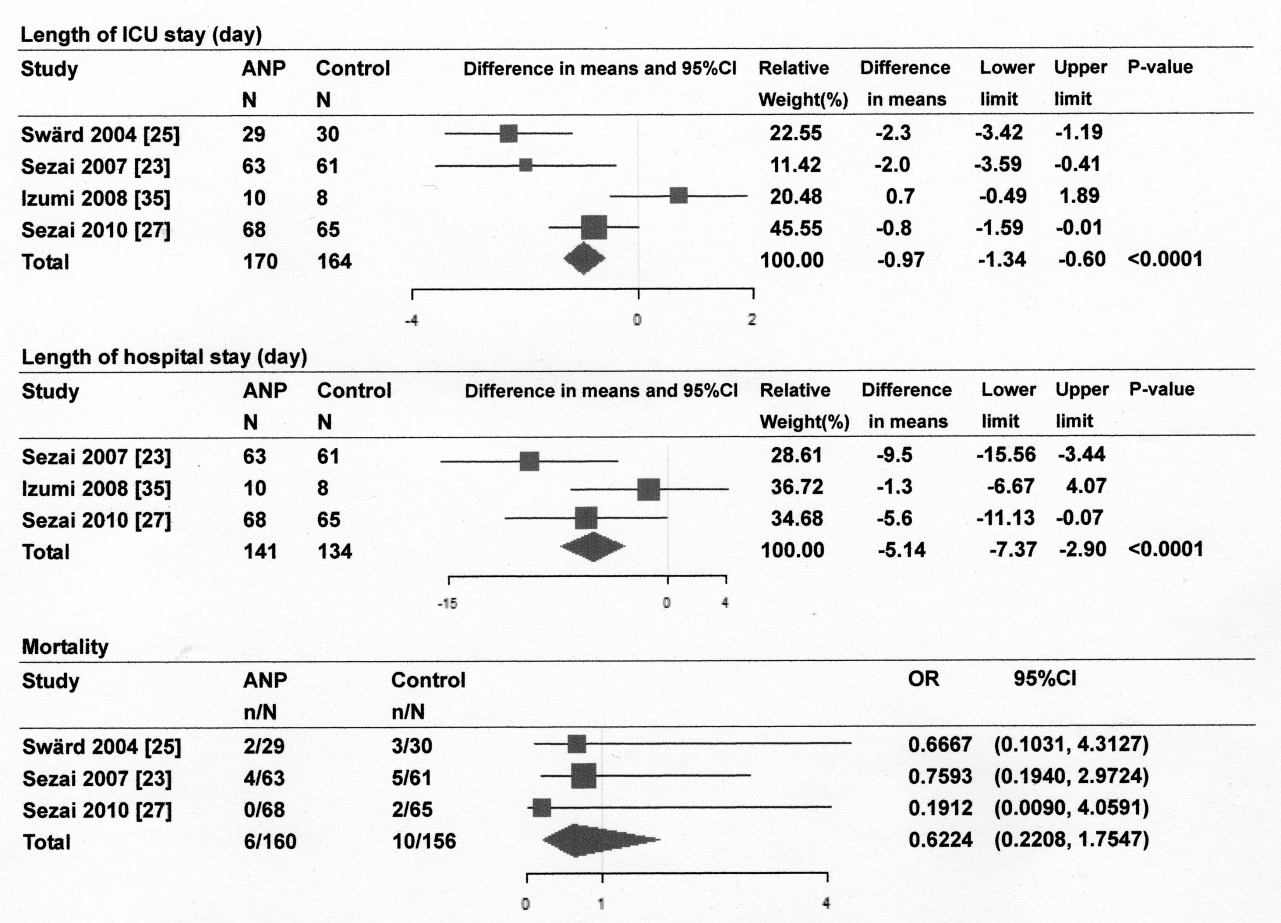
**Effect of atrial natriuretic peptide (ANP) infusion on length of ICU stay and hospital stay and mortality**. Size of data markers is proportional to the weight of each study in the forest plot. N, number of patients; Horizontal bars = 95% confidence interval (CI).

**Figure 4:**
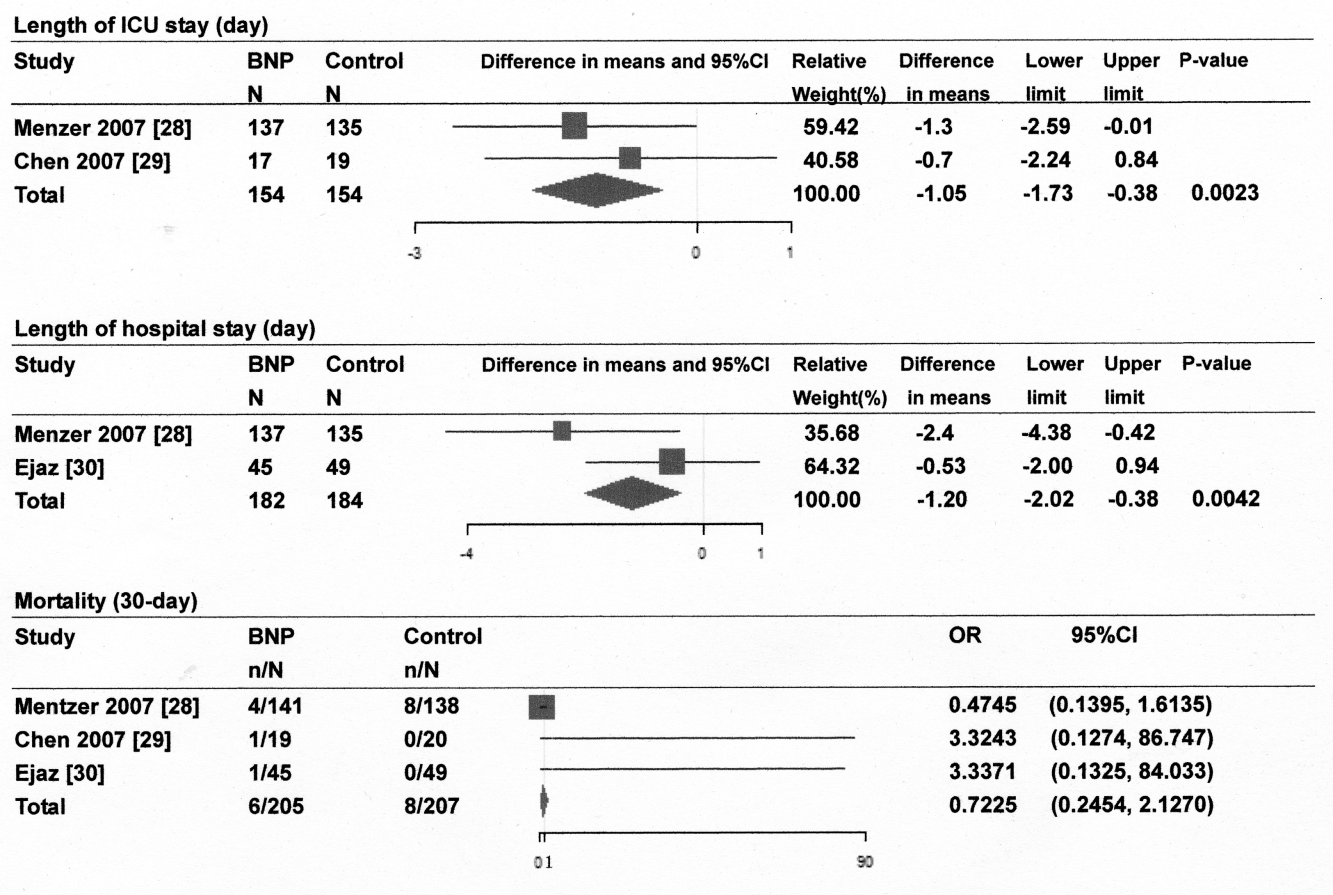
**Effect of B-type (or brain) natriuretic peptide (BNP) infusion on length of ICU stay and hospital stay and mortality**. Size of data markers is proportional to the weight of each study in the forest plot. N, number of patients; Horizontal bars = 95% confidence interval (CI).

Of the 893 references screened, 15 randomized controlled trials (RCTs) of the infusion of ANP or BNP in cardiovascular surgery patients were included in systematic review and 9 studies for meta-analysis (Figure 1). Most of the studies described single-center studies with small samples sizes. Tables 1 and 2 show the respective effects of ANP and BNP on hemodynamics, renal function, and other parameters in cardiovascular surgery patients. The methodological quality of the included studies was as follows. The patients were randomly allocated in all studies. Allocation concealment was adequate in 11 studies [21-31], which blinded the participants and the investigators, and unclear in the remaining studies. Four studies [[25,27] to [29]] excluded withdrawals after randomization, but the remaining eleven studies did not have withdrawals. Completeness of follow up was adequate in all studies. There was no relationship between funding source and ANP or BNP in these studies. The main results of the meta-analysis were as follows: ANP infusion significantly decreased peak serum creatinine levels, incidence of arrhythmia and need for renal replacement therapy (Figure 2), and ANP or BNP infusion significantly decreased the length of ICU stay and hospital stay compared with controls, but not mortality compared with controls (Figures 3 and 4).

**Table 1:** Effects of ANP on hemodynamics, renal function, and other parameters in patients undergoing cardiovascular surgery

Author, year, journal, country and study type	Patient group		Surgical procedure and dose of ANP	Effects of ANP on hemodynamics, renal function, and other parameters
	ANP (n)	Control (n)		
Bergman A, *et al*. [22], 1996 J Cardiothorac Vasc Anesth, Sweden, RCT	15	15	Elective CABG on CPB, normal renal function ANP 7.5 pMol/kg/min, after operation for 3 hrs,	MAP→, HR→, PCWP→, RAP→, CI↓ Urine output↑, inulin clearance↑, FF↑, FENa↑
Hayashida N, et al. [34], 2000 Ann ThoracSurg, Japan, RCT	9	9	Elective mitral valve surgery on CPB ANP 0.05 μg/kg/min, after initiation of CPB for 6 hrs	PCWP↓, RAP↓, SVR↓, CI↑, Urine output↑, FENa↑, plasma cGMP↑, plasma BNP↓,
Sezai A, *et al*. [21], 2000 Ann Thorac Surg, Japan, RCT	20	20	Elective CABG on CPB. ANP (SBP > 120 mmHg,0.05 μg/kg/min; SBP ≤ 120 mmHg, 0.03 μg/kg/min), after initiation of CPB for 24 hrs →0.02 μg/kg/min for 4 hrs,	CVP↓, SVRI↓, PVRI↓, plasma cGMP↑, Urine output↑, GFR↑, renin↓, angiotensin II↓, aldosterone↓, pleural effusion↓, furosemide↓
Hayashi Y, *et al*.[32], 2003 ASAIO Journal, Japan, RCT	14	16	Selective open heart operation on CPB ANP 0.025 μg/kg/min, after CPB for 72 hrs,	CVP↓, MPAP↓, PCWP↓, CI↑, furosemide↓, KCL↓, renin↓, aldosterone↓
Swärd K, *et al*. [25], 2004 Crit Care Med, Sweden RCT	29	30	Cardiac surgery, preoperative normal renal function, SCr > 50%↑, ANP 50 ng/kg/min→SCr decreased below the trigger value or RRT	Ccr↑, incidence of renal RRT on day 21 (ANP group 21% versus Control group 47%)↓ RRT-free survival at day 21↑
Sezai A, *et al*. [23], 2007 Circ J, Japan, RCT	63	61	Emergent CABG on CPB ANP 0.02 μg/kg/min at the start of CPB→0.01 μg/kg/min for 12 hrs	Peak CK-MB↓, arrhythmias↓, plasma BNP↓, furosemide↓, KCL↓
Izumi K, *et al*. [35], 2008 Ann Thorac Cardiovasc Surg, Japan, RCT	10	8	Elective cardiac surgery on CPB, SCr ≥ 1.2 mg/dl ANP 0.02 μg/kg/min, for 5 days or more	Urine output↑, SCr↓, Ccr↑, urinary NAG↓
Mitaka C, *et al*.[24], 2008 Crit Care Med, Japan RCT	20	20	Elective abdominal aortic aneurysm repair, SCr < 3 mg/dl ANP 0.01-0.05 μg/kg/min, before aortic cross clamping →for 48 hrs	Urine output↑, SCr↓, Ccr↑, BUN↓, urinary NAG/Cr↓, plasma ANP ↑, plasma BNP ↓, furosemide↓
Sumi K, *et al*. [33], 2008 J Cardiothorac Vas Anesth, Japan, RCT	30	15	Infrarenal abdominal aortic aneurysmectomy ANP 0.02 and 0.05 μg/kg/min, respectively, 5 min after aortic clamping→end of operation	MAP→, MPAP↓, PVRI↓, SVRI→
Sezai A, *et al*. [26], 2009 J Am Coll Cardiol, Japan RCT	251	253	Elective CABG on CPB, SCr < 1.3 mg/dl, Ccr ≥ 80 ml/min, ANP 0.02 μg/kg/min, at the start of CPB→0.01 μg/kg/min for 12 hrs	SCr↓, Ccr↑, SCr > 2.0 mg/dl (ANP group; *n *= 1, Control group; *n *= 8), RRT (ANP group; *n *= 0, Control group;*n *= 4)
Sezai A, *et al*. [27], 2010 J Am Coll Cardiol, Japan RCT	68	65	Cardiac surgery on CPB, LVEF ≤ 35%, ANP 0.02 μg/kg/min, at the start of CPB→0.01 μg/kg/min for 12 hrs	LVEF↑, eGFR↑, SCr↓, plasma BNP↓, arrhythmias↓, mortality (NS), cardiac death-free rate at 5 or 8 years (ANP group; 98.5%, control group; 85.5%)

AKI, acute kidney injury; ANP, atrial natriuretic peptide; BNP, B-type (or brain) natriuretic peptide; BUN, blood urea nitrogen; CABG, coronary artery bypass graft; cGMP, cyclic guanosine, 3',5'-monophosphate; CI, cardiac index; CPB, cardiopulmonary bypass; CVP, central venous pressure; Ccr, creatinine clearance; eGFR, estimated glomerular filtration rate; FF, filtration fractional; FENa, fractional sodium excretion; GFR, glomerular filtration rate; HR, heart rate; LVEF, left ventricular ejection fraction; MAP, mean arterial pressure; MPAP, mean pulmonary arterial pressure; NAG, N-acetyl-beta-D-glucosaminidase; n, number; NS, not significant; PCWP, pulmonary capillary wedge pressure; PVRI, pulmonary vascular resistance index; RAP, right atrial pressure; RCT, randomized controlled trial; RRT, renal replacement therapy; SCr, serum creatinine; SVR: systemic vascular resistance, SVRI: systemic vascular resistance index.

**Table 2:** Effects of BNP on hemodynamics, renal function, and other parameters in patients undergoing cardiovascular surgery

Author, year, journal, country and study type	Patient group		Surgical procedure and dose of BNP	Effects of BNP on hemodynamics, renal function, and other parameters
	BNP(n)	Control(n)		
Mentzer RM, *et al*. [28], 2007 J Am Coll Cardiol, USA, RCT	137	135	CABG on CPB, LVEF ≦ 40%, BNP 0.01 μg/kg/min, after anesthesia for 24-96 hrs	PAP (NS), urine output during the initial 24 hrs (BNP group, 2926 ± 1179 ml vs. Control group 2350 ± 1066 ml, *P *< 0.001), peak increase in SCr (BNP group, 0.15 vs. Control group, 0.34, *P *< 0.001), max decrease in GFR (BNP group, -10.8 ml/min/1.73 m^2 ^vs. Control group, -17.2 ml/min/1.73 m^2^, *P*= 0.001), length of hospital stay↓, 180-day mortality↓
Chen HH, *et al*. [29], 2007 Circulation, USA RCT	20	20	Cardiac surgery on CPB, Ccr < 60 ml/min, BNP 0.005 μg/kg/min, after anesthesia for 24 hrs	Ccr↑, plasma cystatin↓, plasma BNP↑, plasma cGMP↑, aldosterone↓
Beaver TM, *et al*. [31], 2008 J Card Surg, USA RCT	9	10	Maze and mitral valve surgery, BNP 0.01 μg/kg/min, 3 hrs after CPB for 72 hrs	Urine output (NS), plasma ANP↓, furosemide doses (NS), time to extubation (NS), PaO_2_/F_I_O_2 _at 48 hrs (NS)
Ejaz AA, *et al*. [30], 2009 J Thorac Cardiovasc Surg, USA RCT	45	49	High-risk cardiac surgery, BNP 0.01 μg/kg/min, before surgery→for 5 days	SCr ↓, GFR↑, incidence of AKI ↓, incidence of RRT (NS), all cause of mortality through day 21 (NS), length of hospital stay (NS)

AKI, acute kidney injury; ANP, atrial natriuretic peptide; BNP, B-type (or brain) natriuretic peptide; CABG, coronary artery bypass graft; Ccr, creatinine clearance; cGMP, cyclic guanosine 3',5'-monophsphate; CPB, cardiopulmonary bypass; GFR, glomerular filtration rate; LVEF, left ventricular ejection fraction; n, number; NS, not significant; PAP, pulmonary arterial pressure; RCT, randomized controlled trial; RRT, renal replacement therapy; SCr, serum creatinine

### ANP studies

ANP infusion decreased the mean pulmonary arterial pressure [32,33], pulmonary capillary wedge pressure [32,34], central venous pressure or right atrial pressure [21,32,34], systemic vascular resistance index [21,34], and pulmonary vascular resistance index [21,33], but did not change the heart rate or mean arterial pressure [22,33]. There is some disagreement in the reported effects of ANP on cardiac index. Bergman *et al*. [22] showed a decrease in cardiac index after ANP infusion, whereas Hayashi *et al*. [32] and Hayashida *et al*. [34] showed an increase in cardiac index. A meta-analysis showed that ANP infusion significantly decreased the incidence of arrhythmias compared with controls [23,27] (Figure 2). ANP infusion increased urine output [21,22,24,34,35], increased creatinine clearance or glomerular filtration rate (GFR) [21,22,25-27,35], decreased serum creatinine levels [24,26,27,35], and decreased the furosemide dosage [21,24,32], compared with controls. ANP infusion also decreased the incidence of renal replacement therapy compared with controls [25,26]. The meta-analysis showed that ANP infusion significantly decreased peak serum creatinine levels and the need for renal replacement therapy compared with controls (Figure 2). ANP infusion increased plasma cyclic guanosine 3',5'-monophosphate (cGMP) levels [21,34] but decreased plasma BNP levels [23,24,27], compared with controls. ANP infusion decreased renin and aldosterone [21,32] and pleural effusion [21] compared with controls. ANP infusion increased renal replacement therapy-free survival at 21 days compared with controls [25]. The meta-analysis showed that ANP infusion significantly reduced the length of ICU stay and hospital stay, but not mortality compared with controls (Figure 3).

### BNP studies

BNP infusion did not change the pulmonary arterial pressure [28]. Over the first 24 hours after surgery, BNP infusion significantly attenuated the peak increase in serum creatinine, significantly attenuated the fall in GFR, and increased urine output, compared with controls [28]. BNP infusion decreased plasma cystatin levels and maintained creatinine clearance, compared with controls [29]. BNP infusion decreased the incidence of AKI, decreased serum creatinine levels, and increased GFR, compared with controls, but there were no differences between the BNP and control groups in mortality or the incidence of renal replacement therapy [30]. In one study, the urine output and furosemide dosage were not significantly different between the BNP and control groups [31]. BNP infusion increased plasma cGMP levels [29] and decreased plasma ANP levels [31] compared with controls. Time to extubation and PaO_2_/F_I_O_2 _ratio at 48 hours were not significantly different between the BNP group and the control group [31]. One study showed a shorter ICU stay and reduced 180-day mortality in the BNP group compared with the control group [28]. The meta-analysis showed that BNP infusion significantly reduced the length of ICU stay and hospital stay, but not mortality compared with controls (Figure 4).

## Discussion

AKI after cardiac surgery is associated with an increased risk of morbidity, mortality, and length of ICU and hospital stay [4-6]. In a retrospective study of 2,973 patients, long-term mortality after cardiothoracic surgery was proportional to severity of AKI, as defined by RIFLE (Risk, Injury, Failure, Loss, and End stage) classification [36]. In addition, duration of AKI is associated with long-term mortality after cardiac surgery [37]. Therefore, severity and duration of AKI have important implications for patient care. The predominant causes of AKI in patients undergoing cardiovascular surgery are hypoperfusion and inflammation because of cardiopulmonary bypass (CPB). The nonpulsatile flow during CPB has been shown to cause AKI by inducing vasoconstriction and ischemic renal injury [38]. There appear to be other mechanisms responsible for AKI, however, as even patients who undergo surgery off CPB (off-pump) are at risk for AKI. The pathophysiology of AKI is multifactorial. Cardiac-surgery-associated AKI is caused by exogenous and endogenous toxins, metabolic factors, ischemia and reperfusion, neurohormonal activation, inflammation, and oxidative stress [39]. Similar to cardiac surgery, AKI is the most common complication in patients undergoing pararenal aortic aneurysm repair. Suprarenal aortic cross-clamping is associated with a significant postoperative decline in renal function [40]. Even infrarenal aortic cross-clamping is associated with an increase in renal vascular resistance and a decrease in renal blood flow [41]. Therefore, in addition to cardiac surgery, we have included abdominal aortic aneurysm repair as one of cardiovascular surgery. The risk of AKI can therefore be reduced by maintaining adequate hemodynamics and an adequate metabolic state, avoiding nephrotoxic drugs, and inhibiting neurohormonal activation and inflammation during perioperative periods.

Furosemide is frequently used to facilitate fluid and electrolyte management of AKI. However, meta-analysis has shown that furosemide is not associated with any significant clinical benefits in the prevention and treatment of AKI and high doses may even be detrimental [42].

The cardiovascular effects of ANP or BNP are vasodilation and a reduced sympathetic tone in the peripheral vasculature [9]. ANP and BNP bind to natriuretic peptide receptor A, which increases cGMP and dilates veins and arteries [9], and thereby reduces preload and afterload. ANP, meanwhile, decreases pulmonary capillary wedge pressure, central venous pressure, mean pulmonary arterial pressure, pulmonary vascular resistance index, and systemic vascular resistance index [21,32-34]. The ANP patient group also had a reduced incidence of arrhythmias compared with controls [23,27]. The reduction of arrhythmia may be attributable to stable serum potassium levels, as the dose of furosemide and rate of KCl correction are lower in the ANP groups than in the controls [23,32]. However, arrhythmias may be caused by many different factors, including hypomagnesemia, myocardial injury from surgical handling, inflammation after cardiac surgery, acute atrial enlargement from volume overload, inadequate cardioprotection during CPB, and hyperadrenergic state. Although the true mechanism is unknown, we surmise that the anti-arrhythmic effects of ANP are due to prevention of volume overload and suppression of the sympathetic nervous system except for potassium levels.

The natriuretic and diuretic actions of ANP and BNP are caused by renal hemodynamic and direct tubular actions [9]. ANP increases transcapillary filtration pressure within the glomerulus [10], and this then translates to an increase in GFR. The protective effect of ANP for the kidney may take place via the ANP-induced increases in the medullary vasa recta blood flow [43], an action that may protect against medullary ischemia. In one study, the urinary N-acetyl-β-D-glucosaminidase (NAG)/creatinine ratio, an index of renal tubular damage, was significantly lower in the ANP group than in the control group [24].

ANP or BNP infusion increased urine volume, creatinine clearance and GFR and decreased serum creatinine in patients with normal renal function [22,26]. ANP or BNP infusion also attenuated serum creatinine increase and maintained creatinine clearance and GFR in patients with preoperative renal dysfunction [35], in patients with AKI after cardiac surgery [25] and in patients with left ventricular ejection fraction (LVE F) ≤40% [28]. Especially, Mentzer *et al*. [28] have shown that the beneficial effects of BNP on postoperative renal dysfunction were prominent in patients with renal dysfunction at baseline serum creatinine (Cr) > 1.2 mg/dl. In these patients, BNP significantly inhibited serum creatinine increase and GFR decrease compared with those of normal renal function. Therefore, the renal effects of ANP and BNP may depend on degree of underlying renal function.

Although 10 of 15 studies did not mentioned fluid balance, some of the studies suggest that the control groups were given more volume. This may be relevant to recent studies on AKI and volume loading [44], which have shown that adequate volume repletion is of major importance for prevention of AKI.

ANP and BNP also suppress the renin-angiotensin-aldosterone axis [9], thereby counterbalancing the vasoconstrictive effects of these neurohormones. In fact, two clinical studies have shown that ANP infusion decreases renin and aldosterone compared with controls [21,32]. The effects of ANP in preserving the peritubular capillary blood flow and suppressing the renin-angiotensin system may protect against renal tubular damage after cardiovascular surgery. The cardiovascular and renal effects of ANP and BNP and the action of these natriuretic peptides in manipulating the renin-angiotensin-aldosterone axis may help to prevent serious AKI in the management of patients with cardio-renal syndrome.

The major site of ANP synthesis is the atrium, and ANP mRNA levels are considerably higher in the atrium than in the ventricles. However, ventricular ANP expression is re-induced in heart failure. Although concentrations of BNP and BNP mRNA are much lower in the ventricle than in the atrium, the total content of BNP and BNP mRNA in the ventricle accounts for 30% and 70% of that in the whole heart, respectively. In patients with congestive heart failure, concentrations of myocardial BNP mRNA and circulating BNP are increased more than are those of ANP, suggesting that BNP plays the role of an emergency hormone against ventricular overload. The characteristic feature of BNP mRNA that is different from ANP mRNA is a conserved sequence consisting of repeated AUUUA units in the 3' untranslated region. The presence of this sequence accelerates the degradation of BNP mRNA. Therefore, BNP gene expression is regulated differently from ANP gene expression and is thought to dynamically change, depending on the physiological and pathophysiological conditions [45]. While ANP and BNP seem to play discrete pathophysiological roles in a dual natriuretic peptide system [14,15], their roles may also be compensatory in relation to each other. ANP and BNP bind to the same natriuretic peptide receptor A [9] and this may lead to mutual biological invasion of ANP and BNP. ANP infusion decreases plasma BNP levels in patients undergoing cardiovascular surgery, while BNP infusion decreases plasma ANP levels in the same patients [23,24,31,34]. This tells us that ANP and BNP are closely related.

There are many natriuretic peptide receptors A in the body. If there are some unoccupied natriuretic receptors A on the various target cells, exogenous ANP (or BNP) may bind to these receptors, leading to biological effects. In fact, in patients with high plasma BNP levels (> 230 pg/ml), the effect of ANP infusion on left ventricular contractility was blunted but its beneficial effects on left ventricular diastolic function and left ventricular-arterial coupling remained [46]. Thus, ANP infusion may improve left ventricular diastolic function even in patients with high plasma levels. Taken together, extra ANP (or BNP) are likely to confer benefit, even if the endogenous system is at full capacity.

### Limitations of this meta-analysis

First, few studies use the same protocols and the end points are vastly different. The primary endpoints in most of the studies were continuous changes in serum creatinine and creatinine clearance or GFR. The clinically important endpoints of renal replacement therapy and/or mortality were considered primary outcomes in only seven of the studies [23,25-30], and most of the studies lacked the robustness to demonstrate beneficial effects of ANP or BNP on mortality. Second, the included studies had patients with different renal functions and differed significantly in doses, timing of initiation and duration of ANP or BNP treatments. These facts restrict comparing these studies with each other and the reliability of our findings. Third, since there were no standard indications for renal replacement therapy, the decision to initiate renal replacement therapy was dependent on the participating doctors. Therefore, there may be a wide variation in the incidence of renal replacement therapy. Fourth, four out of eleven studies were performed by the same group of investigators in the same institution. Accordingly, the results may be biased more slightly toward the studies of this group. Finally, as a marker of renal function there are some shortcomings of creatinine because serum creatinine is affected by age, gender, muscle mass, nutritional status and analytical interference, although AKI has been defined largely by changes in creatinine. Estimated GFR formulae based on serum creatinine, age, sex and weight are used in daily clinical practice, but their accuracy is debatable. In addition, a 24 hour urine creatinine clearance has been commonly employed for the detection of AKI. However, since a substantial fraction of urinary creatinine is derived from tubular secretion, creatinine clearance regularly overestimates GFR [47]. Accordingly, we have to interpret the results carefully by considering the shortcomings of creatinine.

Even so, ANP and BNP have cardiovascular and renal effects as well as effects in manipulating the renin-angiotensin-aldosterone axis. On these grounds, we speculate that ANP and BNP may be able to prevent or treat for AKI in cardiovascular surgery patients. The infusion of ANP or BNP in these patients should be investigated more thoroughly.

## Conclusions

The infusion of ANP or BNP may preserve renal function after cardiovascular surgery. Only seven studies have researched the incidence of renal replacement therapy and the longer-term outcome in patients after cardiovascular surgery. The meta-analysis showed that ANP infusion significantly decreased peak serum creatinine levels, incidence of arrhythmia and renal replacement therapy, and ANP or BNP infusion significantly decreased the length of ICU stay and hospital stay compared with controls. A large, muticenter, prospective, randomized controlled trial will have to be performed to assess the therapeutic potential of ANP or BNP in preventing and treating AKI in the cardiovascular surgical setting.

## Key messages

• Data on infusion of ANP and BNP in cardiovascular surgery patients was collected from fifteen RCTs and combined.

• ANP infusion increased urine output and the creatinine clearance or GFR, and reduced the use of diuretics and the serum creatinine levels compared with controls.

•The meta-analysis showed that ANP infusion significantly decreased peak serum creatinine levels, the incidence of arrhythmia and hemodialysis, and ANP or BNP infusion significantly decreased the length of ICU stay and hospital stay compared with controls.

• The combined data were insufficient to determine how ANP or BNP infusion during the perioperative period affect the long-term outcome in cardiovascular surgery patients.

## Abbreviations

AKI: acute kidney injury; ANP: atrial natriuretic peptide; BNP: B-type (or brain) natriuretic peptide; BUN: blood urea nitrogen; CPB: cardiopulmonary bypass; cGMP: cyclic guanosine 3',5'-monophosphate; GFR: glomerular filtration rate; LVEF: left ventricular ejection fraction; NAG: N-acetyl-β-D-glucosaminidase; RCTs: randomized controlled trials.

## Competing interests

The authors declare that they have no competing interests.

## Authors' contributions

CM conceived the study and wrote the manuscript. TK and GH conceived the study and conducted the literature search. MT performed the meta-analysis. All of the authors read and approved the final manuscript for publication.
